# Integrative Analysis of miRNA-mRNA and miRNA-miRNA Interactions

**DOI:** 10.1155/2014/907420

**Published:** 2014-02-12

**Authors:** Li Guo, Yang Zhao, Sheng Yang, Hui Zhang, Feng Chen

**Affiliations:** Department of Epidemiology and Biostatistics, School of Public Health, Nanjing Medical University, Nanjing 211166, China

## Abstract

MicroRNAs (miRNAs) are small, noncoding regulatory molecules. They are involved in many essential biological processes and act by suppressing gene expression. The present work reports an integrative analysis of miRNA-mRNA and miRNA-miRNA interactions and their regulatory patterns using high-throughput miRNA and mRNA datasets. Aberrantly expressed miRNA and mRNA profiles were obtained based on fold change analysis, and qRT-PCR was used for further validation of deregulated miRNAs. miRNAs and target mRNAs were found to show various expression patterns. miRNA-miRNA interactions and clustered/homologous miRNAs were also found to contribute to the flexible and selective regulatory network. Interacting miRNAs (e.g., miRNA-103a and miR-103b) showed more pronounced differences in expression, which suggests the potential “restricted interaction” in the miRNA world. miRNAs from the same gene clusters (e.g., miR-23b gene cluster) or gene families (e.g., miR-10 gene family) always showed the same types of deregulation patterns, although they sometimes differed in expression levels. These clustered and homologous miRNAs may have close functional relationships, which may indicate collaborative interactions between miRNAs. The integrative analysis of miRNA-mRNA based on biological characteristics of miRNA will further enrich miRNA study.

## 1. Introduction

MicroRNAs (miRNAs) are small (~22 nts) endogenous noncoding RNAs (ncRNAs). They have many biological roles and act by negatively regulating mRNA expression at the post-transcriptional level [1–3]. They suppress gene expression via interaction with their target messenger RNAs (mRNAs) and either block the translation process or initiate cleavage. These small regulators have important roles in multiple essential biological processes, including cell differentiation and apoptosis [4]. They are also involved in pathological processes and contribute to occurrence and the development of some cancers [5–7]. Abnormal expression of the small ncRNAs may lead to cell death or abnormal cell phenotypes via miRNA-mRNA interactions [8]. Specifically, abnormally expressed miRNAs have been shown to be crucial contributors and may serve as biomarkers in many human diseases.

Bioinformatics analysis indicates that a specific miRNA can regulate expression of up to thousand mRNAs through miRNA-mRNA association, and a specific mRNA can be regulated by multiple miRNAs. miRNAs may also be regulated as potential targets *in vivo* [9]. The flexible regulatory pattern should exist between the two coding (mRNA) and noncoding (miRNA) RNA molecules. Numerous reports have shown that miRNA-mRNA interaction is more complex than we had believed, and a series of studies have been performed to predict miRNA-mRNA modules and correlation networks using miRNA and mRNA expression profiles [10–13]. miRNA-miRNA interactions can also be observed between natural sense and antisense miRNAs [14–18]. These miRNAs always more pronounced divergences in expression, because they may complementarily bind and restrict each other. Many miRNAs are not randomly distributed but rather clustered on chromosomes and cotranscribed as a single polycistronic transcript [19, 20]. Some of these clustered miRNAs can be considered homologous miRNAs (members of the same miRNA gene family). Their sequences are more similar to each other than those of other miRNAs. This is especially true of miRNAs with conserved seed sequences (nucleotides 2–8) [21]. These miRNA gene clusters and gene families always have close functional relationships and coregulate or coordinately regulate multiple biological processes [22–24].

Integrative analyses that are based on miRNA-mRNA interactions always aim to develop algorithms or tools [25, 26]. Few studies have addressed the biological characteristics of miRNA in miRNA-mRNA interactions. For example, miRNAs are prone to cluster on the chromosomes, some miRNAs show more sequence similarity than others, and a single miRNA locus can yield a cluster of isomiRs with various 5′ and 3′ ends and length distributions [23, 27, 28]. In the present study, an integrative analysis of miRNA-mRNA was performed based on miRNA and mRNA expression profiles in human HepG2 and L02 cells by applying high-throughput techniques. HepG2 cells are human hepatoma cell lines, and they are a suitable model to study occurrence of development of human hepatocellular carcinoma *in vitro*. L02 is the normal human liver cell line, which is always used as control cell lines of HepG2 cells. The purpose of this study was to improve understanding of miRNA-mRNA interactions in regulatory networks. The patterns of expression of potential miRNA-miRNA pairs were also analyzed comprehensively, and the patterns of expression of miRNAs with potential functional relationships, including members of the same miRNA gene clusters and gene families, were surveyed.

## 2. Materials and Methods

### 2.1. miRNA and mRNA Profiling Using High-Throughput Techniques

HepG2 and L02 cells were obtained from the American Type Tissue Collection. miRNA expression profiles were generated from Illumina Genome Analyzer IIx, and then analyzed using Novoalign software (http://www.novocraft.com/, v2.07011) based on the latest known human pre-miRNAs in the miRBase database (Release 19.0, http://www.mirbase.org/) [29]. To further understand expression patterns of target mRNAs of miRNAs, mRNA expression profiles were assessed using microarray hybridization. Hybridization was performed in Agilent's SureHyb Hybridization Chambers (Human LncRNA Array v2.0, 8 × 60 K, Arraystar).

### 2.2. Data Analysis

**Figure 1:**
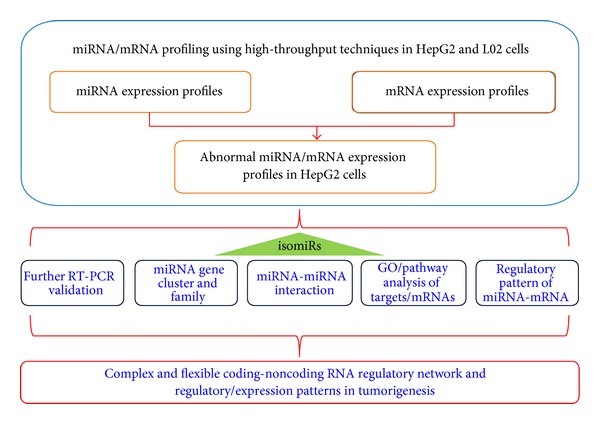
The miRNA-mRNA integrative analysis.

Aberrantly expressed miRNAs and mRNAs in HepG2 cells were surveyed and identified via fold change analysis. To filter out rare species with lower levels of relative expression, fold change values were estimated by adding additional units (10 units). A detailed flow chart showing the integrative analysis of miRNA-mRNA is given in Figure 1. The main steps were as follows: (1) Abnormal miRNAs and mRNAs were first surveyed through bioinformatics analysis. miRNA expression analysis was also performed at the isomiR level, including the different selections of isomiRs (the most abundant isomiR, sum of all isomiRs, and the canonical miRNA sequence) [28]. (2) Several deregulated miRNAs were further experimentally validated using qRT-PCR. (3) The potential expression and functional relationships among miRNAs were evaluated through analysis of the patterns of expression of clustered and homologous miRNAs based on miRNA gene clusters and families. miRNA-miRNA pairs with potential interactions were also screened and analyzed. (4) GO/pathway terms were enriched based on deregulated mRNAs and the target mRNAs of miRNAs, and miRNA-mRNA regulatory patterns were predicted based on expression profiles.

The experimentally validated target mRNAs of those abnormal miRNAs were obtained from the miRTarBase and Tarbase databases [30]. Common target mRNAs were subjected to functional enrichment analysis using CapitalBio Molecule Annotation System V4.0 and compared to abnormally expressed mRNA profiles from microarray datasets (MAS, http://bioinfo.capitalbio.com/mas3/). GO and pathway analyses were used to determine the biological roles of deregulated miRNA and mRNA species. Potential miRNA-mRNA and miRNA-miRNA interactions and miRNA/mRNA expression profiles were used to construct functional interaction networks using Cytoscape v2.8.2 Platform [31].

### 2.3. qRT-PCR Validation

Abnormal miRNAs were further validated using quantitative real-time reverse transcription PCR (qRT-PCR) using SYBR premix Ex Taq (Takara, Japan). Samples were amplified using the Mastercycler ep realplex2 system (Eppendorf, Hamburg, Germany). qPCR was performed using specifically designed primers and used to detect hsa-miR-15b/103a/106b (Bulge-Loop miRNA qRT-PCR Primer Set, RiboBio, Guangzhou, China), and U6 served as an internal control. The relative amount of each miRNA was measured using the 2^(−ΔΔCT)^ method [32]. All qRT-PCR reactions were carried out in triplicate, and data were presented as the mean ± standard deviation. The two-tailed Student's *t* test was used to compare the expression difference between tumor and normal cells.

## 3. Results

### 3.1. Overview of miRNA/mRNA Expression Profiles and Further Experimental Validation

**Figure 2:**
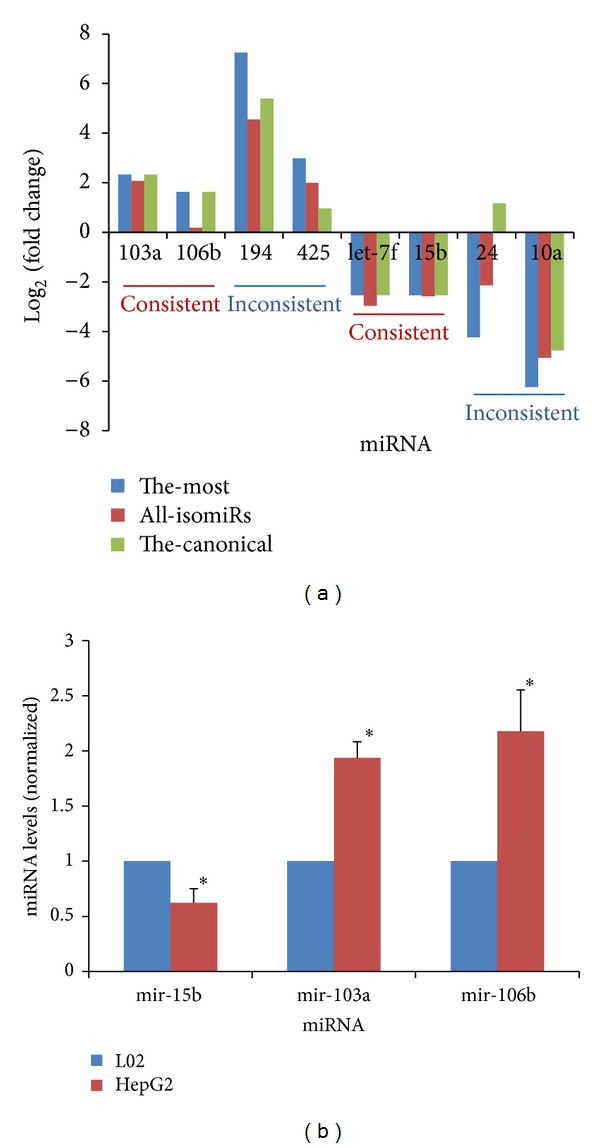
(a) miRNA expression analysis and (b) and further qRT-PCR validation. (a) The fold change values (log 2) differ in the variety of miRNA sequences involved. “The-most” indicates the most abundant and dominant isomiR sequence. “All-isomiRs” indicates sum of all isomiRs. “The-canonical” indicates the reference miRNA sequence in the miRBase database. The canonical miRNA sequence may be consistent or inconsistent with the most abundant isomiR sequence. Different methods of estimation may produce different fold change values (log 2), but they always show consistent deregulation patterns. (b) Further RT-PCR validation is performed for miR-15b, miR-103a, and miR-106b, and the experimental results show consistent deregulation patterns. “*” indicates that the *P* value is less than 0.05.

Upregulated and downregulated miRNAs/mRNAs were identified using the fold change values (log 2) based on the control sample. Many miRNAs and mRNAs were found to be differentially expressed (see Figure S1 in the Supplementary Material available online at http://dx.doi.org/10.1155/2014/907420). miRNA expression patterns were further analyzed at the isomiR level. Fold change values were found to diverge based on the different selections of isomiRs (the most abundant isomiR, sum of all isomiRs, and the canonical miRNA sequence) (Figure 2(a)). Differences in fold change values rarely affected the selection of deregulated miRNA species. The canonical or annotated miRNA sequences were not always the most dominant species in the miRNA locus. They had even lower levels of expression. The qRT-PCR primers used here were designed according to the canonical miRNA sequences in the miRBase database (Release 19.0, http://www.mirbase.org/) [29]. For this reason, in order to further validate deregulated miRNAs using qRT-PCR technique, we randomly selected several abnormally expressed miRNAs (miR-15b, miR-103a, and miR-106b; their canonical miRNA sequences were the most abundant isomiRs) for further experimental validation (Figure 2). Bioinformatic analysis showed that miR-103a and miR-106b were upregulated in tumor cells, while miR-15b was identified as downregulated species (Figure 2(a)). As expected, qRT-PCR experimental validation showed consistent results (Figure 2(b)).

### 3.2. Expression Patterns of miRNA-miRNA Pairs and miRNA Gene Clusters and Families

The expression patterns of miRNA-miRNA pairs that can form miRNA-miRNA duplexes were also analyzed [18]. Eight miRNA-miRNA pairs were found to be abundantly expressed in HepG2 or L02 cells. Expression analysis showed one member of each natural miRNA-miRNA pair to be abundantly expressed and the other to be quite rare (Table 1). For example, the miR-103a/miR-103b pairs showed a pronounced difference in the degree of expression: miR-103a was abundantly expressed (normalized sequence count was more than 9,232 in tumor cells), and miR-103b was not detected. Pronounced differences in degree of expression were quite common between these two members of each miRNA-miRNA pair (Table 1).

**Figure 3:**
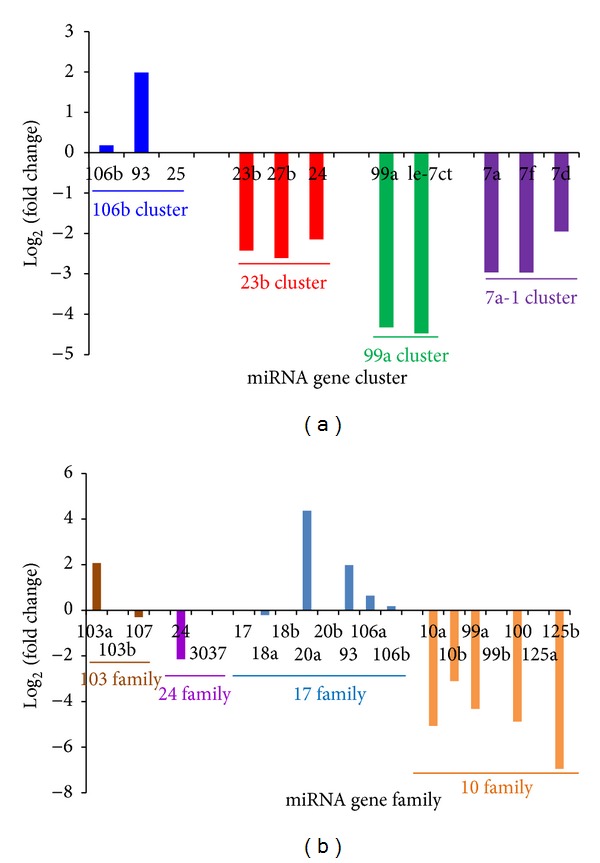
Examples of (a) deregulated miRNA gene clusters and (b) gene families. (a) Clustered and (b) homologous miRNAs are always consistently upregulated or downregulated in tumor cells, although they can differ in fold change values (log 2) and relative expression levels. miRNAs shown here to have zero change (such as miR-25) are not detected or did not show significant differences between tumor and normal cells.

The expression patterns of miRNAs that might have potential functional relationships were also analyzed. Clustered and homologous miRNAs always showed consistent patterns of deregulation (Figure 3), although they could differ in relative level of expression, sometimes showing large differences. These differences in expression may have led to the various fold change values observed between these related miRNA members (Figure 3). For example, the miRNA in the miR-23b gene cluster were downregulated, showing similar fold change values, and those of the miR-106b gene cluster showed highly different fold change values (Figure 3(a)).

### 3.3. Expression and Regulatory Patterns of miRNAs/mRNAs and Functional Enrichment Analysis

**Figure 4:**
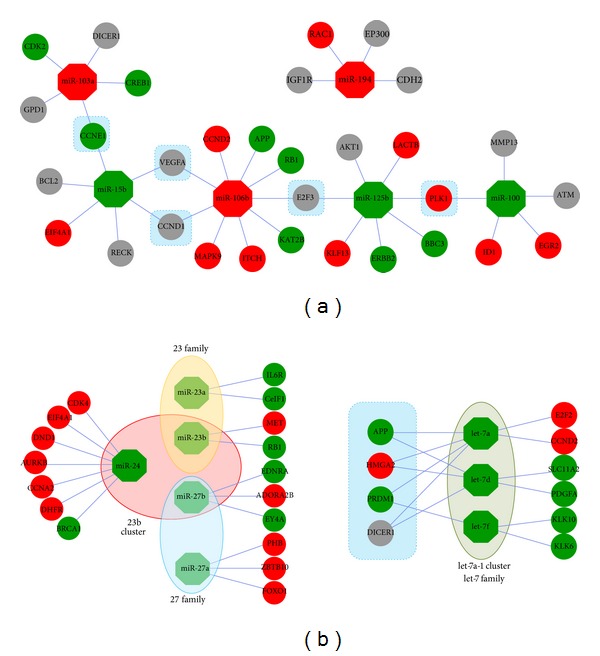
Examples of flexible and selective regulatory network between miRNAs and mRNAs. (a) Selected overexpressed (miR-103a, miR-106b, and miR-194) and underexpressed (miR-15b, miR-100, and miR-125b) miRNAs are used to reconstruct the regulatory network. Their experimentally validated target mRNAs show various expression patterns: some are stably expressed, and others are upregulated or downregulated. Overexpressed miRNAs and mRNAs are here highlighted in red octagons and ellipse, respectively, and underexpressed miRNAs and mRNAs are highlighted in green octagon and ellipse, respectively. Grey ellipses indicate stably expressed mRNAs and mRNAs are not detected in the present study. The targets common to different miRNAs are highlighted in blue rectangles. (b) Selected underexpressed miRNA gene clusters (miR-23b and let-7a-1) and gene families (miR-23 and miR-27) also show complex regulatory networks. These clustered and homologous members are consistently downregulated in tumor cells, and their validated targets show various expression patterns. miRNAs in the let-7a-1 gene cluster are also members of the let-7 gene family. The targets common to these miRNAs have shown upregulated, downregulated, and stable patterns of expression.

Although each aberrantly expressed miRNA can negatively regulate target mRNAs via miRNA-mRNA association, their potential targets always show dramatically different expression patterns (Figure 4). Common target mRNAs might be detected between different deregulated miRNAs, even between upregulated and downregulated miRNAs (according to validated miRNA-mRNA interaction, *E2F3* can be negatively regulated by upregulated miR-106b and downregulated miR-125b, Figure 4). Targets of miRNAs of the same gene clusters and families also showed complex expression patterns, although these related miRNAs were downregulated in tumor cells (Figure 4(b)). These homologous and clustered miRNAs were always simultaneously upregulated or downregulated. They might negatively target the same mRNAs (Figure 4(b)).

Functional enrichment analysis based on the deregulated target mRNAs suggested multiple biological roles (Figures S2, S3, and S4). They were found to contribute to many biological processes, such as the cell cycle, calcium signaling pathway, p53 signaling pathway, and T cell receptor signaling pathway. These aberrantly expressed mRNA species are also involved in some human diseases, including pancreatic cancer, renal cell carcinoma, prostate cancer, and colorectal cancer.

## 4. Discussion

In the study, integrative analysis of miRNA-mRNA is performed using biological characteristic of miRNAs, and miRNA-miRNA interaction is simultaneously analyzed based on the relationships between different miRNAs (Figure 1). Compared to other algorithms or tools of miRNA-mRNA analysis [25, 26], the approach aims to track miRNA-mRNA and miRNA-miRNA interactions based on characteristic of miRNAs. Specifically, (1) miRNAs are prone to detected homologous miRNAs with higher level of sequence similarity, (2) miRNAs are prone to cluster together with close physical distance, (3) some miRNAs are located on sense and antisense strands of specific genomic regions, and (4) miRNA locus can generate multiple isomiRs with various sequences and expression levels, and so forth. Although these specific features of miRNAs have been widely concerned in miRNA study, they are rarely mentioned or involved in miRNA-mRNA analysis. Indeed, many miRNAs coordinately contribute to biological processes, and one specific biological pathway will involved in a series of mRNAs and regulatory miRNAs. Therefore, it is quite necessary to study miRNA-mRNA interactions using characteristic of miRNAs, especially homologous and/or clustered miRNAs are prone to have functional relationships. More importantly, the canonical or annotated miRNA sequence is only one specific member of the multiple isomiRs, and the study at the isomiR level will enrich miRNA study. IsomiR expression patterns contribute to tracking pre-miRNA processing and miRNA maturation processes and understanding regulatory network at the isomiR levels.

According to the integrative analysis method, firstly, aberrantly expressed miRNA and mRNA profiles were collected based on fold change analysis. To further validate these deregulated miRNA species, several deregulated miRNAs that had been experimentally validated using qRT-PCR were randomly selected. As expected, qRT-PCR experiments showed results consistent with those of bioinformatic analysis (Figure 2). As in other reports, miR-103a, and miR-106b were overexpressed in hepatocellular carcinoma (HCC) and served as important negative regulators [33, 34]. However, miR-15b was found to be upregulated [35]. The overexpression of miR-15b may restrict cell proliferation and increase the rate of cellular apoptosis, and abundant expression may indicate a low risk of HCC recurrence [36]. The dynamic expression of miR-15b may play multiple biological roles in tumorigenesis.

Many reports have shown that multiple isomiRs (miRNA variants) can be detected at the same miRNA locus. This is due to imprecise and alternative cleavage of Drosha and Dicer [23, 27, 28]. According to three different methods of estimation methods, the most abundant isomiR, the sum of all isomiRs, and the canonical miRNA, the phenomenon of the multiple miRNA variants may influence the relative expression levels and lead to various fold change values (Figure 2(a)) [24, 28]. This is mainly because of differences among isomiR repertoires and expression patterns, although they are always well conserved across different tissues and animal species [28, 37, 38]. Differences in isomiR expression profiles may play a role in occurrence and development of disease [28]. Generally, consistent deregulated miRNAs could be identified using different methods of estimation methods, even if they have different fold change values (Figure 2(a)). However, if abnormal miRNA expression profiles are collected using the typical methods of analysis of canonical miRNA or the sum of all isomiRs, the difference in fold change values may affect the collection of deregulated miRNA species and may require further experimental validation. Among multiple isomiRs, the canonical miRNAs are not always the most abundant (Figure 2(a)). Some of them can be very rare. Other abundant isomiR species, especially isomiRs with novel 5′ ends and seed sequences (5′ isomiRs), may also be regulatory molecules. These 5′ isomiRs may have novel potential target mRNAs and may contribute to the regulation of previously unknown biological processes. Collectively, it may be best to observe deregulated miRNAs through bioinformatic analysis at the miRNA level using the most abundant and dominant isomiR sequence and isomiR profiles through bioinformatic analysis at the isomiR level based on variations in sequence and expression levels.

miRNAs negatively regulate mRNA expression and contribute to many biological processes through complementary binding to their target mRNAs. Some miRNAs can interact with the 3′-untranslated region (UTR) of target mRNA and reduce the level of mRNA expression [39]. An attempt was here made to reconstruct the coding-noncoding RNA regulatory network according to negative regulation and the deregulation of miRNAs and target mRNAs. Although miRNAs can be either downregulated or upregulated in tumor cells, their experimentally validated and predicted targets may show consistent or inconsistent deregulation patterns (Figure 4). Abnormal miRNA and mRNA expression profiles complicate the regulatory network, although they showed close functional relationships by forming miRNA-mRNA duplexes. A single miRNA can regulate multiple target mRNAs and vice versa. The fact that a single miRNA can engage in many possible miRNA-mRNA interactions can render regulatory networks highly complex. Flexible regulatory patterns indicate that a specific miRNA may regulate selected specific targets and so contribute to specific stages of development. miRNA-mRNA may affect the spatial-temporal expression patterns of miRNAs, but these interactions can also be more strictly regulated during specific stages of development. The selection of regulated target mRNAs may have been driven by functional pressure in cellular environments through complex regulatory mechanisms. In this way, overexpressed, underexpressed, and stably expressed target mRNAs can be identified for specific upregulated and downregulated miRNAs (Figure 4). A single mRNA can be negatively regulated by selected specific miRNAs. The coding-noncoding RNA regulatory network is more complexity than previously thought, especially for complicated and selective multiple interactions of miRNAs and mRNAs (Figure 4).

Functional miRNA groups also contribute to the complexity of regulatory networks. miRNAs that have completely or partially complementary structures can form miRNA-miRNA duplexes through reverse complementary binding events. They can also form miRNA:miRNA* or miRNA-#-5p:mRNA-#-3p duplexes [14, 16–18]. miRNA:miRNA interactions are specific phenomenon. They are especially common between natural or endogenous sense and antisense miRNAs. Possibly because of restricted interactions, these miRNA-miRNA pairs show greater differences in the level expression than other miRNAs do: one member typically has a far higher level of enrichment than the other, which can be quite rare (Table 1). This indicates that restricted interactions may be a regulatory pattern in the miRNA world. Another, very different, type of interaction between miRNAs, termed coordinated interaction, also contributes to the pronounced efficiency of the regulatory process. Some miRNAs, such as clustered and homologous miRNA species, may coregulate or coordinately regulate biological processes [19, 40]. They may be located close to another (clustered in the same genomic region, miRNA gene cluster) or may share sequence similarity (homologous miRNAs, miRNA gene family). Some clustered miRNAs share sequence similarity and are identified as both members of the same cluster and of the same family. These phenomena are not random but rather derived from functional and evolutionary pressures. These related miRNAs always show similar or consistent patterns of deregulation (Figure 3), although they may have different levels of enrichment because of maturation and degradation mechanisms. Deregulation patterns may cause functional relationships. This indicates that collaborative interactions may take place within the coding-noncoding RNA regulatory network. Therefore, related miRNAs further complicate the regulatory patterns, especially when they share specific target mRNAs. In summary, coordinated interactions and restricted interactions both exist in the world of small, noncoding RNA. Although they can be thought of as indirect and direct interactions, respectively, these interactions represent the versatility and complexity of the functional and evolutionary relationships among different miRNAs. miRNA-miRNA interactions enrich and complicate the coding-noncoding RNA regulatory network and contribute to the robustness of the regulatory network in organism.

## Supplementary Material

Scatter plot and hierarchical cluster analyses of mRNA expression profiles are presented in Figure S1. According to the related deregulated miRNAs in Figure 4, gene pathway analysis are presented in Figure S2-S4.

## Figures and Tables

**Table 1:** Differences in expression between natural sense and antisense miRNAs.

miRNA/miRNA	The most abundant isomiR		Sum of all isomiRs		The canonical miRNA	
	HepG2	L02	HepG2	L02	HepG2	L02
103a/103b	9232/—	1833/—	10639/—	2525/—	9232/—	1833/—
122/3591	163/—	938/—	837/—	3550/—	10/—	17/—
203/3545	—/—	216/—	—/—	705/—	—/—	12/—
24/3074	787/2	14998/—	6618/3	29094/—	3592/—	1597/—
423-5p/3184-3p	1208/—	3159/—	1882/—	5790/—	1208/—	3159/—
423-3p/3184-5p	981/—	5036/1	1934/—	8290/1	981/—	5036/—
7-5p/3529-3p	1132/—	939/—	1931/—	2397/—	238/—	386/—
374b-5p/374c-3p	137/—	67/—	318/—	203/—	137/—	67/—

Based on the different methods of estimation, the most abundant isomiR, sum of all isomiRs, and the canonical miRNA, relative expression levels of these pairs of miRNA pairs were determined. They are presented here using normalized data. One member of each pair was always far more abundantly expressed than the other. “—” indicates an undetectable miRNA.
