# Supplementary material for: Integrative Analysis of miRNA-mRNA and miRNA-miRNA Interactions
Source: Biomed Res Int. 2014 Feb 12;2014:907420. doi: 10.1155/2014/907420 (PMC3945032; doi:10.1155/2014/907420)
Supplement: Supplementary file 1 — Scatter plot and hierarchical cluster analyses of mRNA expression profiles are presented in Figure S1. According to the related deregulated miRNAs in Figure 4, gene pathway analysis are presented in Figure S2-S4. [file 907420.f1.pdf]

Supplementary files

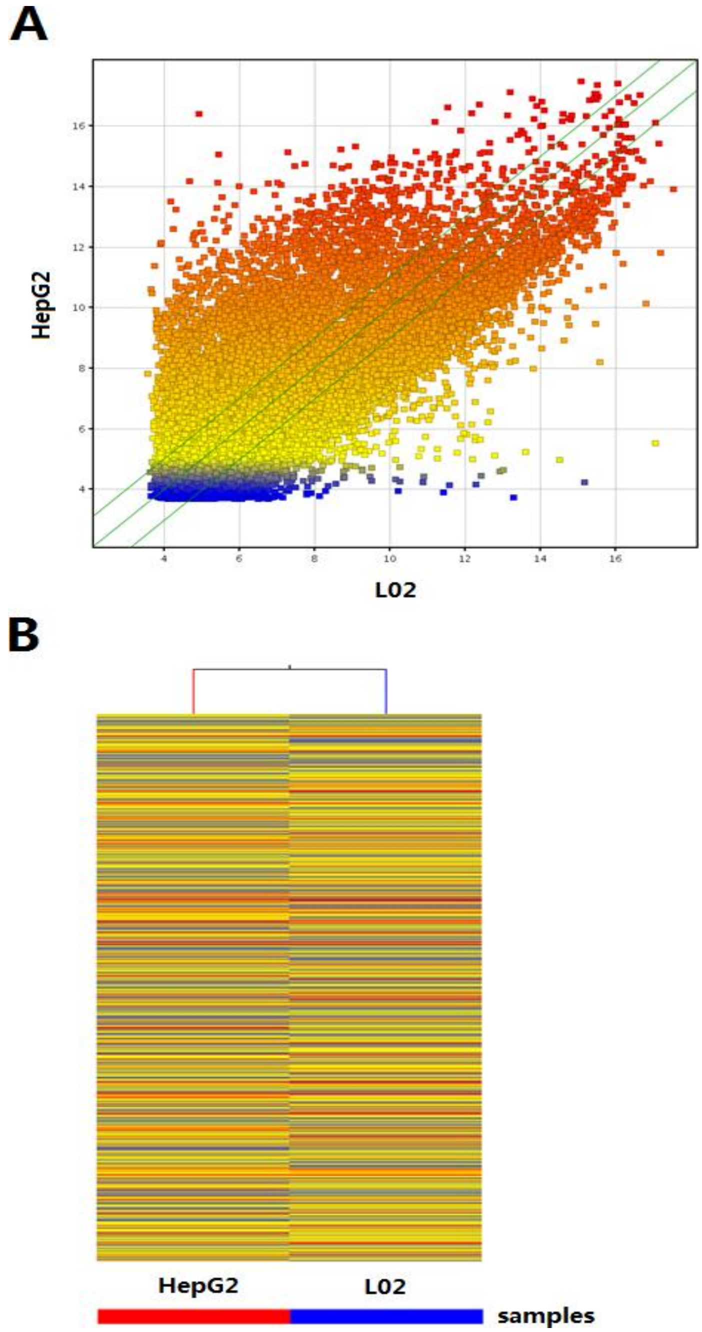

Fig. S1 (A) Scatter plot and (B) hierarchical cluster analyses of mRNA expression profiles in HepG2 and L02 cells.

(A) The scatterplot is a visualization method to assess the variation between tumor and normal cells. The X-axis indicates normal cells (L02), while the Y-axis indicates tumor cells (HepG2). Fold change value of tumor and normal cells are estimated based on normalized data. Different colors of plots indicate relative expression levels, such as blue plots indicate that these mRNAs have lower expression levels in tumor cells. (B) Hierarchical cluster analysis of mRNA expression profiles, which shows different gene expression profiling between tumor and normal cells.

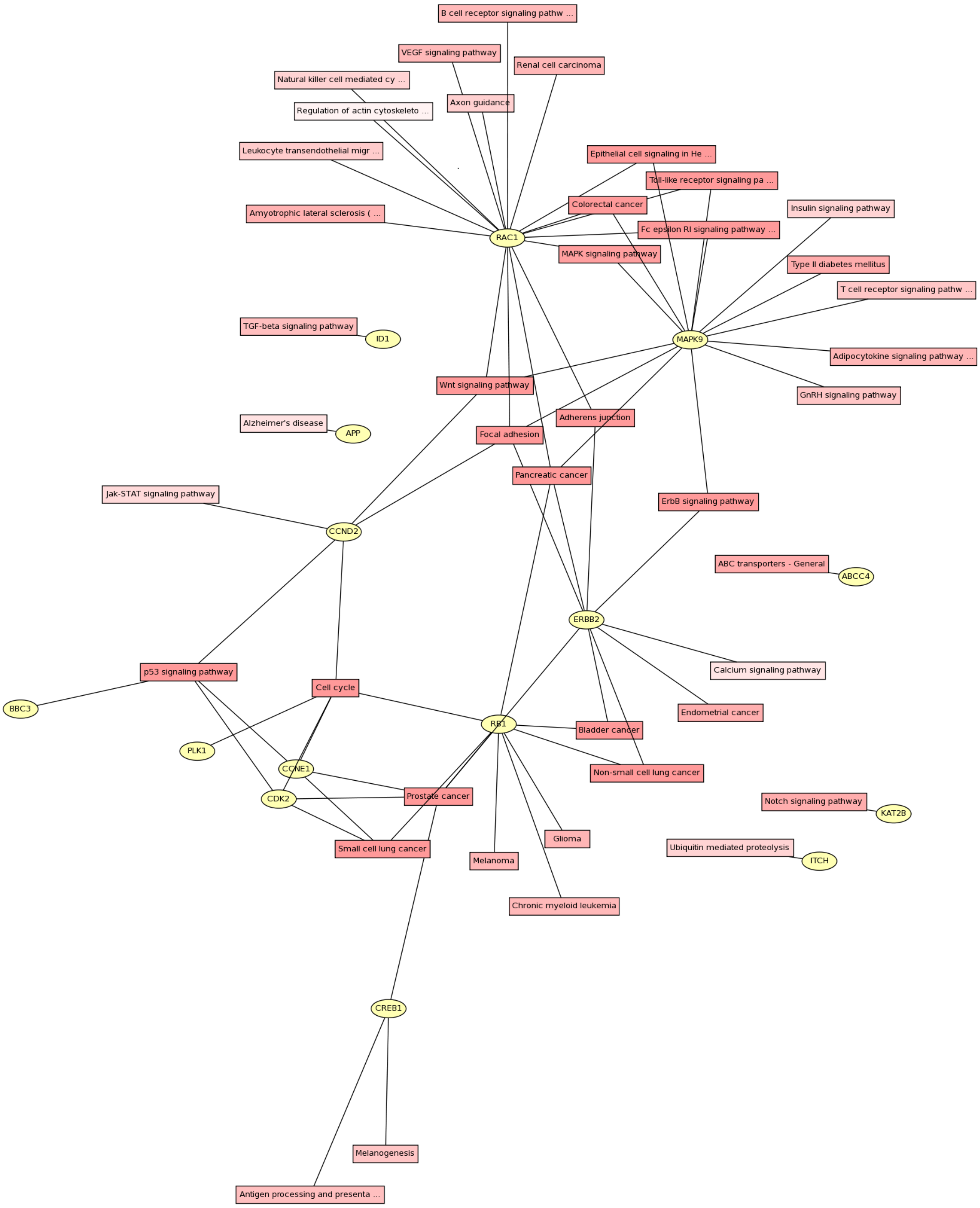

Fig. S2 Gene pathway analysis in Fig. 4(A).

The gene pathway is analyzed based on the KEGG database and on deregulated target mRNAs of miRNAs, as shown in Fig. 4(A).

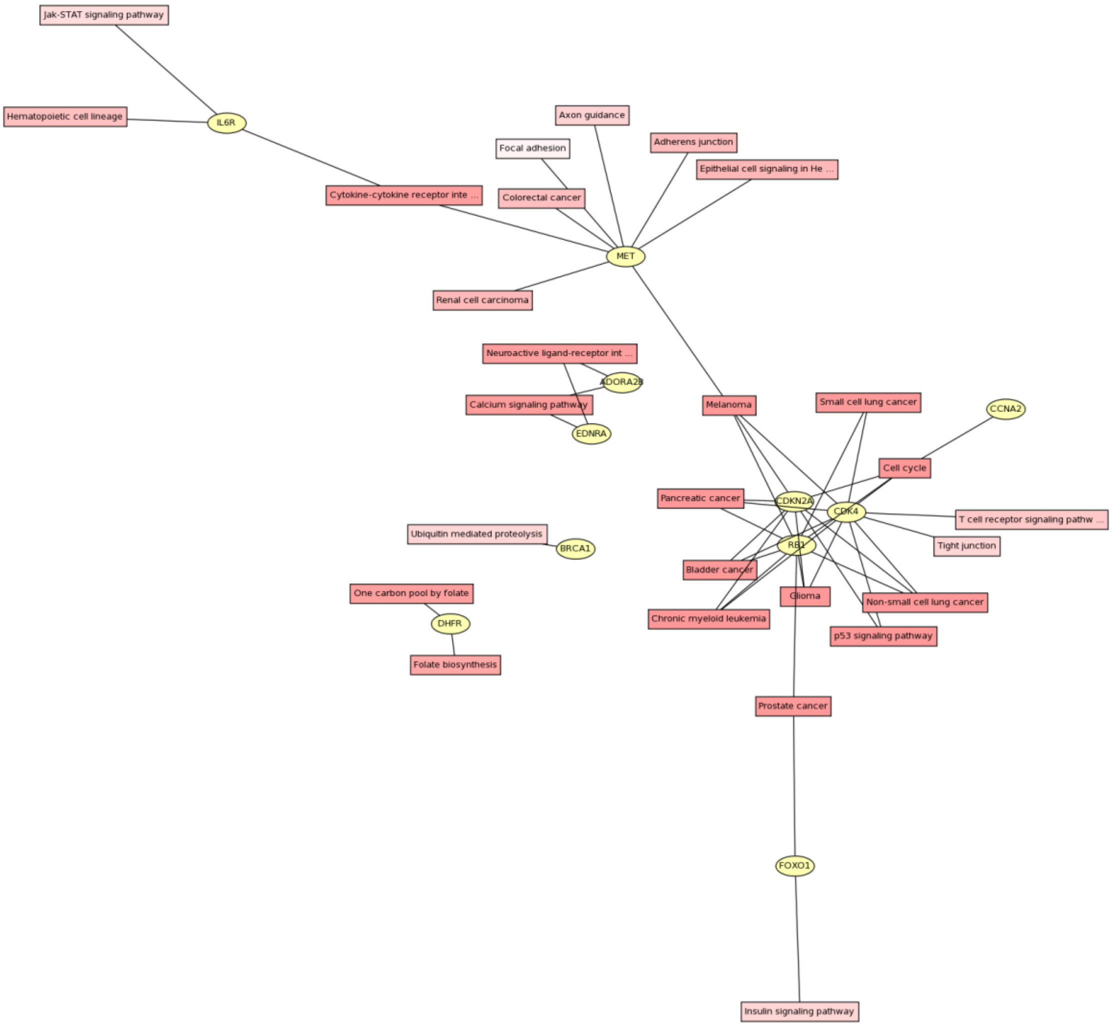

Fig. S3 Gene pathway analysis in Fig. 4(B).

The gene pathway is analyzed based on the KEGG database and on deregulated target mRNAs of miRNAs in Fig. 4(B) (miRNAs in miR-23 and miR-27 gene families and miR-23b gene cluster).

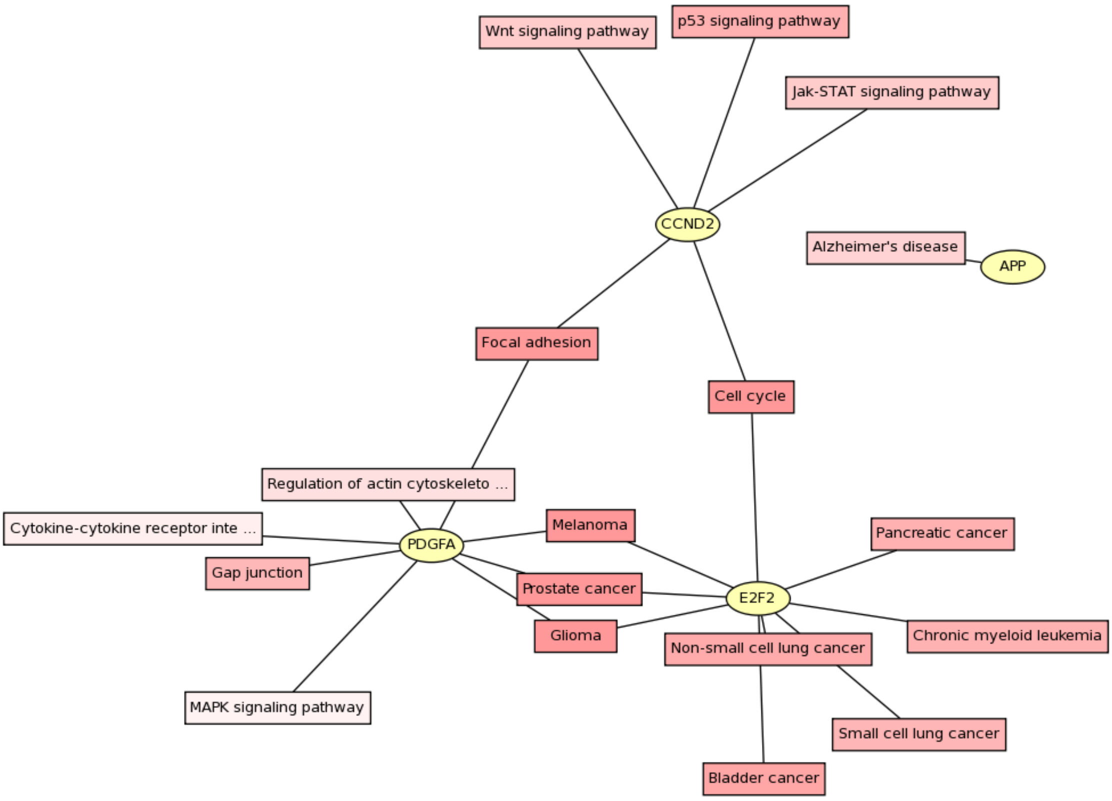

Fig. S4 Gene pathway analysis in Fig. 4(B).

The gene pathway is analyzed based on the KEGG database and on deregulated target mRNAs of miRNAs in Fig. 4(B) (miRNAs in let-7a-1 gene cluster).
